# The Utility of Magnetic Resonance Imaging-compatible Pacemakers in Neurosurgical Patients

**DOI:** 10.7759/cureus.3374

**Published:** 2018-09-27

**Authors:** Gregory Basil, Karthik Madhavan, Ricardo J Komotar, Roger Carrillo, Allan D Levi

**Affiliations:** 1 Neurological Surgery, University of Miami Miller School of Medicine, Miami, USA; 2 Cardiothoracic Surgery, University of Miami Miller School of Medicine, Miami, USA

**Keywords:** magnetic resonance imagining (mri), computed tomography, pacemaker, tumor, abscess

## Abstract

Artificial implantable pacemakers have long been a challenge to neurosurgeons seeking to perform advanced diagnostic imaging on their patients. Unfortunately, while the use of implantable pacemakers has been a life-saving advance for those with cardiac arrhythmias, they also often prevent these patients from undergoing magnetic resonance imaging (MRI). There have been multiple reported cases of pacemaker failure in the context of MRI use. Recent technological advances, however, have allowed the development of pacemakers that are not affected by the MRI scanner. Similar technology has also been applied to the development of MRI-compatible spinal cord stimulators and other neurostimulation devices. In this paper, we discuss four specific neurosurgical cases where the use of MRI was critical for diagnostic and therapeutic decision-making. Current non-MRI-compatible pacemakers were exchanged for MRI-compatible pacemaker technology with some associated cost and risk. The diagnostic cranial and spinal MRIs subsequently obtained were critical for forging the ensuing neurosurgical care. Based on these cases, we extrapolate the importance of MRI-compatible pacemakers to society at large and advocate for the use of such devices in all patients going forward.

## Introduction

The concept of artificial pacing of the heart is one that traces its origins as far back as the French Revolution, where researchers performed electrical experiments on the executed bodies of the condemned [1]. The first true cardiac pacing machines, however, were developed much later, in the 1920s, with a more modern version following shortly after in the 1930s [1]. However, while pacemaker technology has been present for a significant period of time, there has been a steady increase in its utilization over the past 20 years, with a 55% increase in pacemaker procedures observed in Medicare patients from 1990 to 2000 [2]. Additionally, given the pathophysiology of acquired cardiac arrhythmias, pacemakers are far more likely to be placed in the elderly. In fact, while the prevalence in the general population is 2.6 per 100,000, the prevalence in patients 74 years or older jumps to 26 per 100,000 [3]. Indeed, in one epidemiologic study of pacemakers, it was found that approximately 87% of patients with pacemakers were 65 years of age or older [3].

Given the advanced average age of patients with pacemakers, it is not surprising that these patients carry a higher disease burden than the general population. In fact, it has been estimated that 50%-75% of patients living with a pacemaker will require magnetic resonance imaging (MRI) over the course of their life [4]. Unfortunately, and somewhat paradoxically, it is also well established in the literature that conventional pacemakers are not generally compatible with magnetic resonance imaging. The risks of an MRI in patients with pacemakers is based on three main physical interactions between the MRI scanner and the pacing device: the static magnetic field, the pulsed radiofrequency field, and the gradient magnetic field [4]. Each of these forces produces a specific dysfunction, which could lead to serious patient harm and even death. Specifically, MRI can induce currents on implanted leads, potentially causing tissue damage and the induction of tachyarrhythmias. Indeed, one study reports that there have been 17 deaths to date related to MRI scans in patients with pacemaker devices [5]. These risks may also extend to other interventions beyond MRI scans. The effects of a radiofrequency field on pacemaker function have also been discussed in reference to percutaneous trigeminal rhizotomies, where the radiofrequency field could theoretically lead to pacemaker interference [6].

As a result of this problem, there has been a concerted effort by device manufacturers to develop MRI-compatible pacemakers. These MRI-compatible systems have been designed to minimize the impacts of the abovementioned magnetic fields, which can result in device malfunction [7]. Several of these systems have been evaluated in multi-center, randomized controlled trials and have been shown to be safe with no degradation of image quality. Considering the advent of MRI-compatible pacemakers, and the tremendous need for magnetic resonance imaging in the elderly, we believe serious consideration should be given to implanting MRI-compatible pacemakers going forward. In addition, we advocate for the surgical removal of non-MRI-compatible devices and replacement with MRI-compatible alternatives in complex patients (described herein) who present as diagnostic dilemmas with the potential need for neurosurgical intervention. This becomes especially important in patients where an MRI scan could result in a significant impact on the clinical and therapeutic decision-making process. We present four cases of patients with traditional, non-MRI-compatible pacemakers, where we believe MR imaging was critical in driving the surgical decision-making and planning process. As a result, these patients underwent surgical removal of their previous pacemaker and replacement with a new, MRI-compatible alternative.

## Case presentation

Case 1

**Figure 1:**
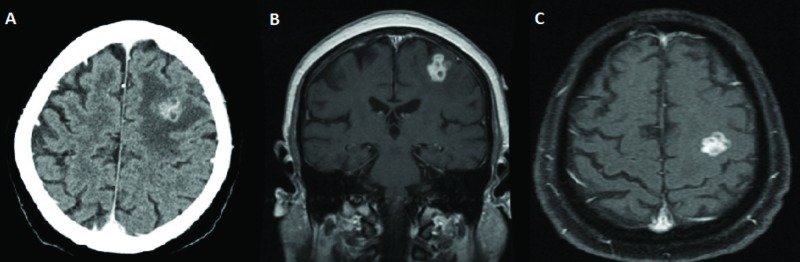
Preoperative imaging demonstrating a multi-lobulated, relatively uniformly contrast-enhancing lesion in the left frontal lobe just anterior to the motor cortex. A) Axial CT with contrast; B) Coronal T1 MRI with contrast; C) Axial T1 MRI with and without contrast CT: computed tomography; MRI: magnetic resonance imaging

A 67-year-old male with a past medical history of congestive heart failure, non-ischemic dilated cardiomyopathy, atrial fibrillation, and pacer-dependent heart block, presented to the University of Miami emergency department with refractory complex partial seizures. Due to the fact that the patient was pacemaker-dependent, he was unable to undergo MRI. Therefore, a contrasted CT scan of the brain was performed, which demonstrated a heterogeneously enhancing left frontal mass just anterior to the left motor cortex thought to be either a primary central nervous system tumor or a metastatic lesion (Figure 1).

A full metastatic workup was conducted with no primary lesion detected. Given the location of the lesion, further imaging was thought to be necessary for two reasons: 1) to further elucidate the differential diagnosis of the lesion and 2) to help safely guide resection or biopsy of a lesion in close proximity to eloquent brain tissue. As a result, cardiothoracic surgery was consulted for the possible replacement of the patient’s current pacemaker with an MRI-compatible alternative. The patient subsequently underwent the removal of their pacemaker and replacement with a Surescan Medtronic DDD Pacemaker RVDR01 (Minneapolis, MN, US). Following this procedure, an MRI was performed and demonstrated a multi-lobulated, relatively uniformly enhancing left frontal lesion (Figure 1). Due to the lack of a definitive diagnosis, the decision was made to perform an awake craniotomy with MRI-assisted stereotactic guidance and intraoperative electrocorticoraphy for a definitive diagnosis. The lesion was able to be resected with the initial pathology suggestive of *Mycobacterium tuberculosae* brain abscess. The patient was placed on anti-tuberculosis medication and intravenous (IV) antibiotics and discharged home. Approximately one year later, he returned to the hospital after suffering a partial seizure with subsequent right upper extremity weakness. He was found to have a recurrence of his left frontal lesion and was again taken to the operating room for surgical resection. Repeat imaging at one year did not demonstrate any further recurrence of his previously seen lesion.

Case 2

**Figure 2:**
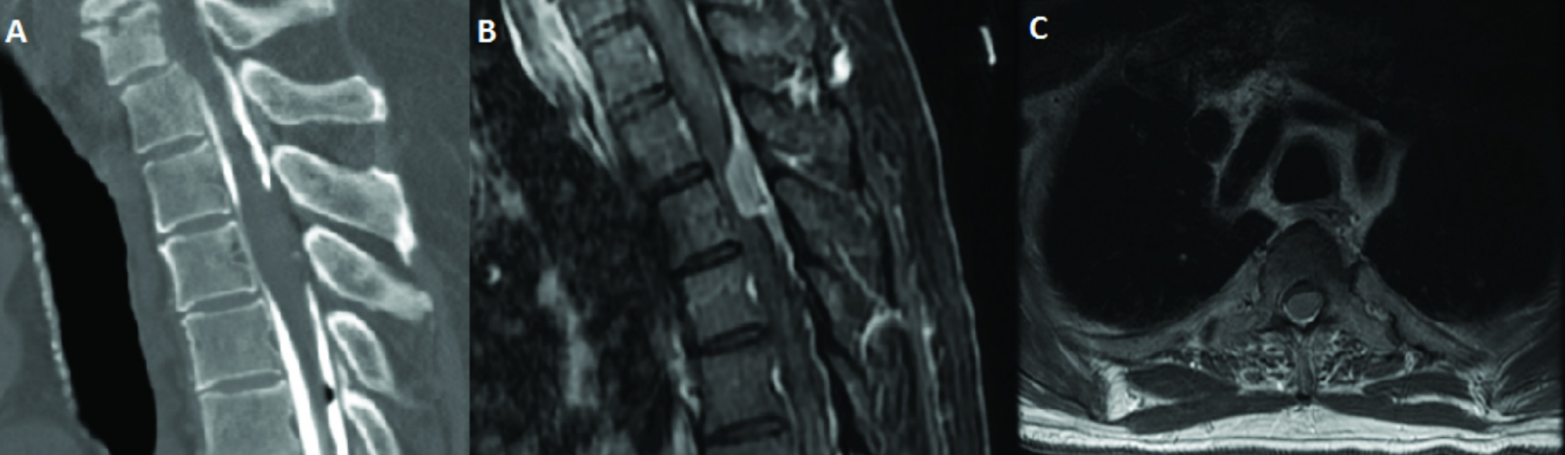
Preoperative imaging. A) Sagittal view of a CT myelogram demonstrating what appears to be an intramedullary lesion at the T3 level; B) Sagittal; C) Axial T1 MRI with contrast of the thoracic spine demonstrating a dorsal, extramedullary dural-based lesion at T3 causing significant canal stenosis and cord compression CT: computed tomography; MRI: magnetic resonance imaging

A 75-year-old male with a past medical history of sick sinus system status post the implantation of a pacemaker in 2004 presented to our service with complaints of chronic numbness in his lower extremities beginning approximately two years prior. He was initially diagnosed with peripheral neuropathy. His numbness was insidious in onset, and he initially presented on his bilateral feet. Over time, the patient’s symptoms progressed to the point where he would experience bilateral thigh numbness, which worsened with ambulating and, subsequently, lower abdominal numbness and urinary retention. His physical exam at that time demonstrated hyper-reflexia in his lower extremities bilaterally along with decreased hip flexor strength and bilateral Babinski signs. Due to concern for myelopathy and the inability to perform an MRI given the patient’s pacemaker, a CT myelogram was performed and demonstrated a likely intramedullary lesion at the T3 level (Figure 2).

The quality of the CT was sub-optimal due to part of the dye being injected in a subdural location. A positron emission tomography (PET) computed tomography (CT) was subsequently performed, which seemed to confirm that the lesion was neoplastic and likely intramedullary in location. Given the advanced age of the patient and multiple comorbidities, further clarity on the nature of the lesion was needed in order to drive a treatment plan and, as a result, cardiothoracic surgery was consulted for the replacement of the pacemaker with an MRI-compatible alternative. The patient subsequently underwent the removal of their pacemaker and replacement with a DDD Medtronic Advisa SureScan pacemaker (Minneapolis, MN, US). Subsequent MRI demonstrated that the lesion was in fact not intramedullary, but presented as a dorsal, extramedullary dural-based lesion at T3 causing significant canal stenosis and cord compression, likely representing a meningioma (Figure 2). Given the location and suspected pathology, it was felt to be surgical despite the patient’s advanced age and comorbidities. The patient subsequently underwent T2-T4 laminectomy with resection of meningioma. At three months follow-up, a repeat MR with gadolinium demonstrated complete resection of the tumor and the patient remained clinically improved two years post-resection with no evidence of recurrence.

Case 3

**Figure 3:**
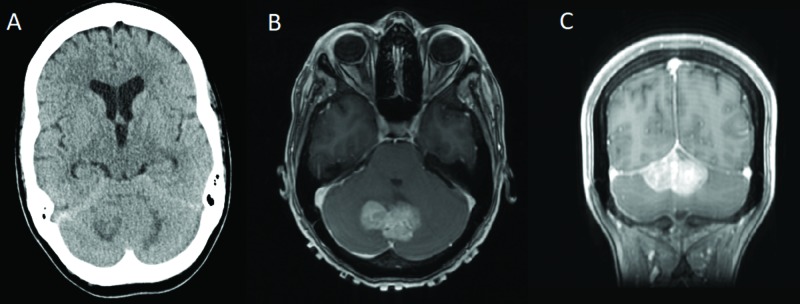
Preoperative imaging. A) Axial CT without contrast demonstrating a midline, superior cerebellar lesion with early hydrocephalus; B) Axial T1 MRI with contrast demonstrating lobulated, enhancing, likely extra-axial mass with low signal on T1; C) Coronal T1 MRI with contrast demonstrating lobulated, enhancing, likely extra-axial mass with low signal on T1 Figure 3A and Figure 3B republished with permission from Chica J, Yepes I, Burks SS, Komotar R, Carrillo R. Case of an intracranial malignant peripheral nerve sheath tumor in the setting of pacer-dependent heart block. Asian Journal of Neurosurgery, Volume 13, Issue: 1, Pages 147-149. Wolters Kluwer- Medknow CT: computed tomography; MRI: magnetic resonance imaging

A 38-year-old female [8] with a past medical history of Hodgkin lymphoma, treated with chemotherapy and radiation, along with radiation-induced heart conduction defect status post pacemaker placement, presented to a hospital in Mexico with a chief complaint of occipital headaches, dizziness, dysarthria, and imbalance. As a result, a CT scan was performed and demonstrated a midline superior cerebellar mass with surrounding edema, early hydrocephalus, and a small tentorial subdural hematoma (Figure 3).

The patient was transferred to our service for further workup and treatment. At the time of her initial presentation, the lesion seen on the CT scan was favored to represent metastatic disease with lymphoma high on the differential diagnosis. Due to the presence of a non-MRI-compatible pacemaker, an MRI was unable to be performed at that time and a full metastatic workup was otherwise unrevealing. Ultimately, given the uncertainty regarding the pathology of the lesion and, as a result, the optimal treatment modality, the decision was made to replace the patient’s pacemaker with a DDD Medtronic Advisa SureScan MRI-compatible pacemaker. Postoperatively, an MRI of the brain was performed, which demonstrated an enhancing, likely extra-axial mass favored to represent a meningioma. As a result, the patient was taken to the operating room for a suboccipital craniotomy. A gross total resection was achieved and pathology returned as a high-grade peripheral nerve sheath tumor. Unfortunately, the patient developed a postoperative hematoma in her surgical cavity, which required emergent evacuation and the placement of an external ventricular drain and, ultimately, a ventriculoperitoneal shunt. The patient, however, made a full recovery and was discharged with plans for adjuvant chemotherapy and radiation.

Case 4

**Figure 4:**
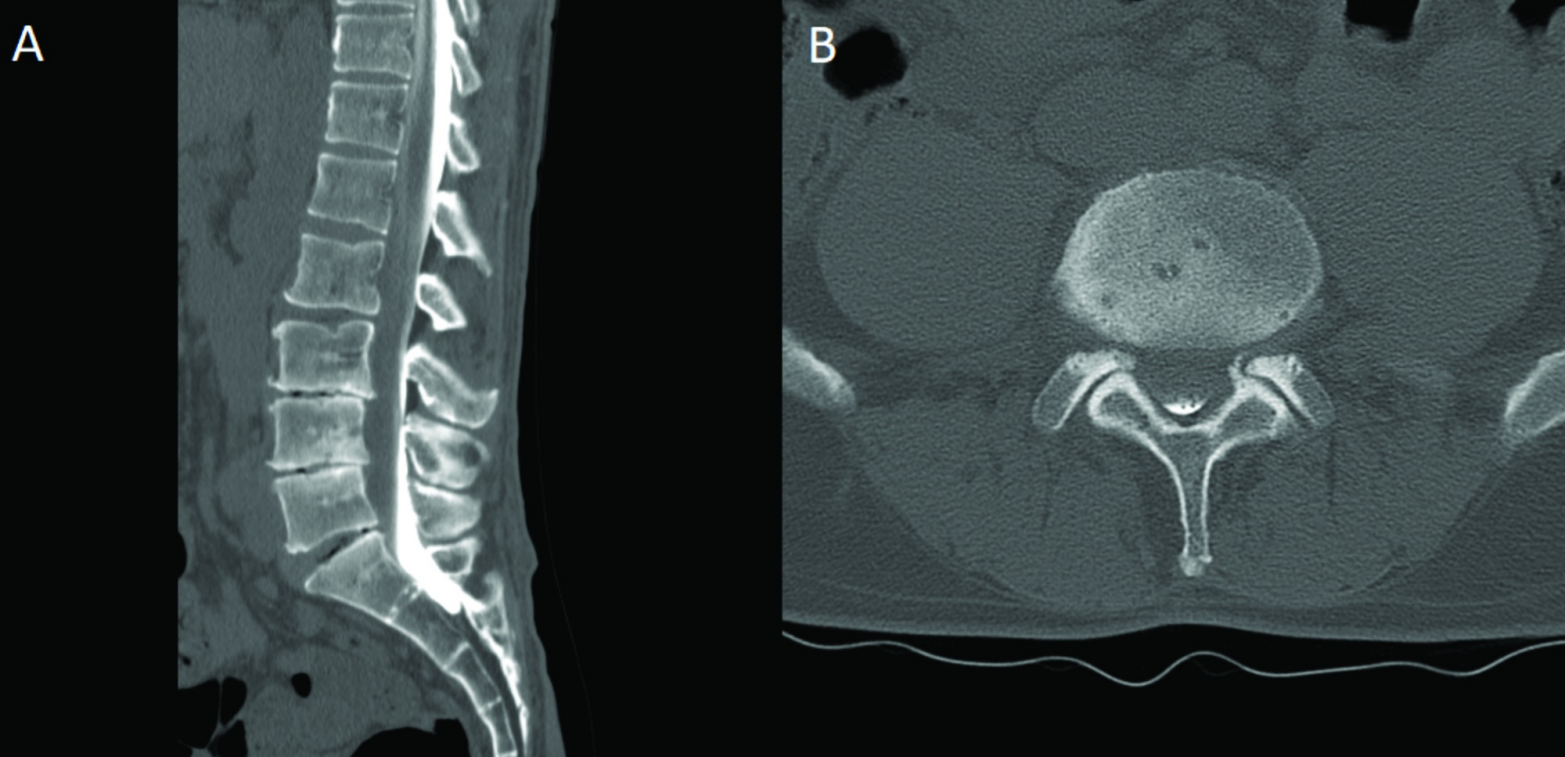
CT myelogram. A) Sagittal cut demonstrating multi-level spondylosis with collapse of disk height most pronounced at L3-L4 and L4-L5; B) Axial cut at the L4-L5 level demonstrating difficulty in determining central or neuroforaminal stenosis using this technique CT: computed tomography

A 50-year-old male presented with a chief complaint of chronic back pain since 1997. He had a history of lumbar surgeries, including a right-sided L4-L5 and L5-S1 hemilaminectomy in 1999. The patient reported that after a car accident in the distant past, he “lost feeling” in his right leg and was told he had severe nerve pain and damage. His chief complaint during his visit to our office was a weakness in his left quadriceps along with back and leg pain and numbness. On physical exam, the patient had subtle weakness in the left hip flexor and left quadriceps, as well as the extensor hallucis longus and the gastrocnemius on the right. He had decreased pinprick in the L3 dermatome on the left but sensation otherwise appeared grossly intact. Deep tendon reflexes were 1+ to 2 throughout bilaterally. Nerve conduction studies revealed axonal loss and motor neuropathy of the peroneal nerve on the right along with chronic irritation of the right L5 nerve root but were otherwise unrevealing. Unfortunately, the patient had a non-MRI-compatible pacemaker, which prevented him from undergoing an MRI. A plain CT of the lumbar spine demonstrated multi-level spondylosis with severe loss of disk height at L3-L4, L4-L5, and L5-S1. In lieu of an MRI, a CT myelogram was performed (Figure 4), which was inconclusive but suggested possible multi-level neural foraminal stenosis at L3-L4, L4-L5, and L5-S1. Notably, a majority of the contrast was located dorsally, which may have been related to operator technique.

**Figure 5:**
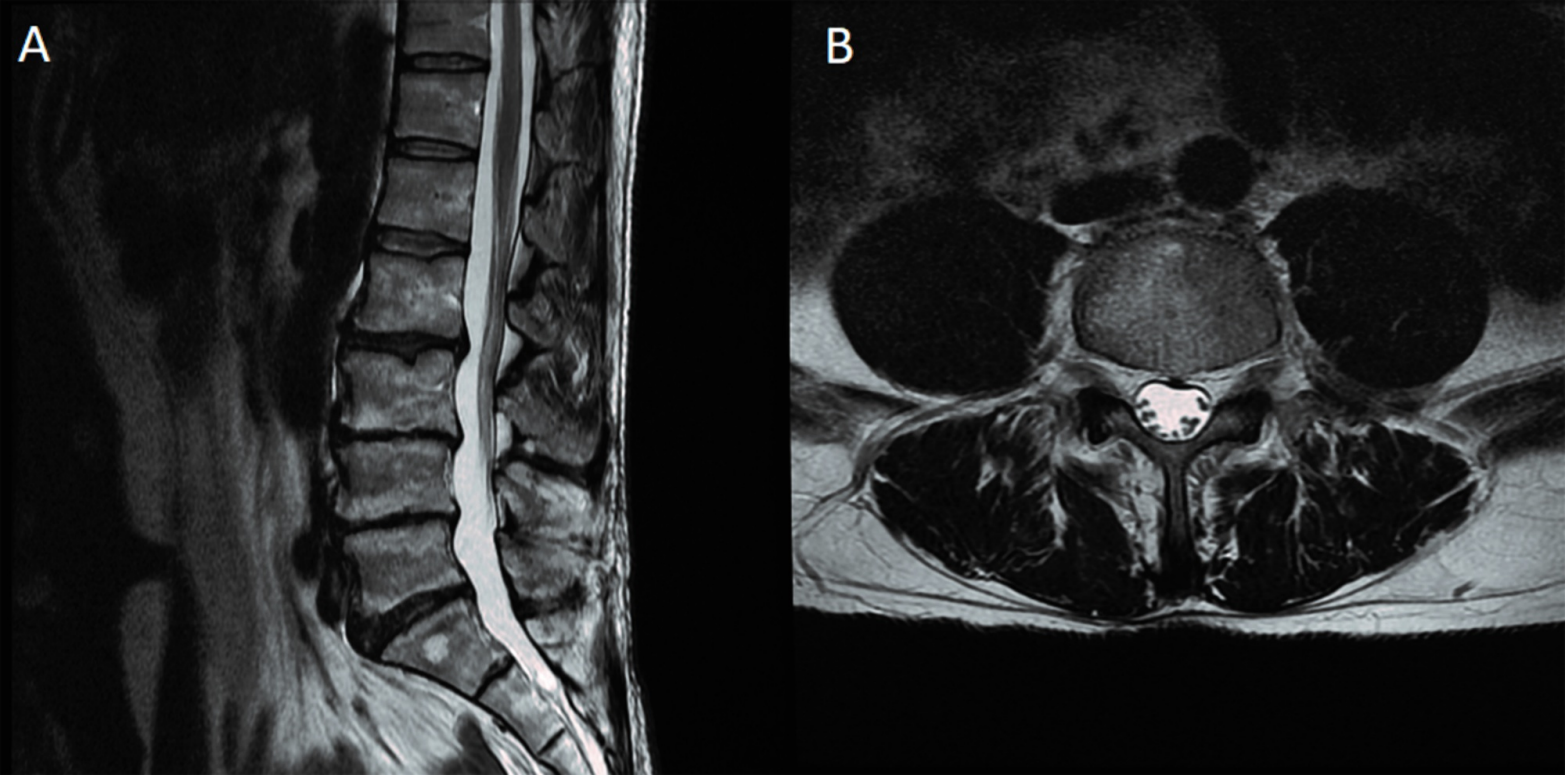
T2 MRI. A) Sagittal cut clearly showing no significant central stenosis; B) Axial cut at the L4-L5 level demonstrating no evidence of neuroforaminal stenosis at this level MRI: magnetic resonance imaging

The patient indicated that he would not be interested in undergoing a large fusion surgery, and so we discussed the possibility of performing selective nerve root blocks, and if these were effective, foraminotomies to offer some symptomatic relief for his leg pain. However, given progressively worsening leg pain and a lack of adequate imaging and unrevealing nerve conduction studies, we requested that the patient have his pacemaker changed with an MRI-compatible alternative to enable the performance of an MRI scan. At that time, the patient was already scheduled for a battery change and, therefore, elected to undergo full replacement of his pacemaker with an MRI-compatible alternative. Following the completion of this surgery, an MRI was performed, which while demonstrating multi-level spondylosis, did not show any significant central canal or neuroforaminal stenosis (Figure 5). As a result, we advised the patient that while his axial back pain may be improved by a fusion surgery, foraminotomies were unlikely to result in any significant symptomatic relief.

## Discussion

MRI is an extremely useful and important imaging modality commonly utilized by neurosurgeons for the last 30 years. The image quality and anatomical definition of the brain, spinal cord, and peripheral nerves are far superior to computed tomography (CT). In addition, by using software modifications, MRIs can provide data on brain chemistry (eg., choline peak – MR spectroscopy), cerebrospinal fluid flow (Cine MR), brain and spinal cord vascularity (magnetic resonance angiogram (MRA) or magnetic resonance venography (MRV)), important regional differences in brain function (functional MR), and, more recently, can provide a non-invasive technique of measuring intracranial pressure [9].

In this paper, we present four cases of patients with conventional non-MRI-compatible pacemakers who required MRI scanning for diagnostic and therapeutic purposes. In all cases, the patient outcome and surgical decision-making process were significantly impacted by the MRI results. In the first case, the MRI scan aided in preoperative planning and facilitated stereotactic guidance for an awake, open surgery. In the second case, while the lesion was initially thought to be intramedullary based on the CT myelogram, a subsequent MRI demonstrated an extramedullary, dural-based lesion, amenable to a straightforward surgical resection with low risk of morbidity. In our third case, while the CT scan suggested an intra-axial cerebellar mass, the MRI scan favored an extra-axial mass, significantly changing preoperative planning. In the final case, while the patient presented with complaints suggestive of radiculopathy, MR imaging clearly demonstrated no significant neural compression, leading us to advise the patient that a decompressive surgery alone was unlikely to provide significant symptomatic relief. These cases serve as an example of the indispensable importance of MRI in modern neurosurgery. Unfortunately, however, the surgeon is often limited in their ability to perform an MRI based on the presence of metallic components in the patient, including shrapnel, artificial valves, pacemakers, spinal cord stimulators, and implanted defibrillators. While some of these factors such as the presence of shrapnel are beyond the surgeon’s control, we propose that the presence of an artificial pacemaker should no longer serve as a barrier to obtaining appropriate imaging.

As with any clinical decision, the benefits of placing a new pacemaker must, of course, be weighed against the risks. In one prospective study, which studied the risk of pacemaker replacement at 72 different academic and private practice centers, the overall major complication rate from replacement without the addition of new transvenous leads is at 4.0% and the complication with the placement of new leads at 15.3% [10]. In the case of the patients requiring new leads, the major complication was the need for reoperation due to lead malfunction or inappropriate lead position [10]. This relatively high rate of complications suggests the need for a clear understanding of risks versus benefits in any case where patients may require an MRI. In the cases we present in this series, we believe that the benefits outweighed the risks, as the patients' clinical course was significantly impacted by their imaging findings. Nevertheless, this decision must not only lie in the hands of the clinician but, more importantly, the patient. The potential benefits of neurosurgical intervention must be carefully weighed against the possibility of complications related to exchanging the existing pacemaker system.

While pacemakers have historically been a relative contraindication to MR imaging, the technology now exists to safely perform magnetic resonance imaging in patients with modern pacemakers designed expressly for this purpose [11-12]. Indeed, the first device of its kind, produced by Medtronic, received FDA approval in 2011, with several others receiving more recent approval, including similar devices produced by Biotronik and Boston Scientific [13]. These devices have not only been proven to be safe from an imaging perspective but also to carry no additional statistically significant surgical risks when compared to conventional pacemakers [14]. Given these readily available alternatives and their comparable safety profile to conventional pacemakers, we propose that going forward, all patients requiring pacemakers be implanted with MRI-compatible devices thus eliminating any potential future conflict between MR imaging and their implanted device. Additionally, in cases where a neurosurgeon deems MR imaging essential for critical clinical decision-making, patients with existing, non-MRI-compatible devices should have their devices replaced with an MRI-compatible alternative with a clear understanding of the risks and potential benefits. These suggestions hold especially true in the population of patients over 65 years of age, who not only comprise the vast majority of patients with pacemakers but also a population of patients who will likely require an MRI in their lifetime. Previous papers have suggested the selective implantation of MRI-compatible devices in patients with certain criteria such as previous MRI and known comorbidities like malignancy, neurologic disorder, planned surgery, or contrast allergy [15]. However, we maintain that given that the disease burden in the predominant age group needing pacemakers, along with the difficulty in predicting future malignancy or need for surgery, a more practical approach and, indeed, long-term, cost-effective approach would be to make MRI-compatible pacemakers the standard for all patients.

Needless to say, our proposal must be supported by a clear understanding of the costs of MRI-compatible pacemakers compared to traditional, non-MRI-compatible pacemakers. It is important to note that the new MRI-compatible devices do come at a premium when compared to the traditional devices. The exact differential in price is difficult to determine, as it will vary based on hospital and vendor, and in cases where the pacemaker must be explanted and replaced, the cost of surgery must also be considered. While these costs undoubtedly add to a large sum on a population level, they cannot be considered in isolation but must instead be compared to the cost of not implanting such devices. Namely, the cost to the healthcare system of patients not receiving the necessary treatments or receiving un-indicated surgeries or procedures due to the inability to make an appropriate diagnosis using MRI technology.

In the four cases presented in this paper, our patients would have had significantly different clinical courses had they not received an MRI. In the first case, MR imaging allowed for stereotactic guidance, which assisted in operative planning, specifically in avoiding damage to the nearby eloquent brain. In the second case, our elderly patient would likely have not received a life-altering surgery had we accepted his CT myelogram studies as the benefit of resecting an intramedullary lesion and its attendant risks of dorsal column dysfunction would have outweighed any potential benefits. This likely results from the fact that with the advent of MR and the subsequent marked reduction in the number of myelograms performed annually – the technical proficiency of imaging specialists in obtaining diagnostic quality myelogram-CTs has likewise declined. This worsened proficiency undoubtedly leads to diagnostic dilemmas, as was seen in the case described above. In the third case, we again demonstrate the diagnostic benefit along with the value in surgical planning of MR imaging. Finally, in the fourth case, our patient was spared an unnecessary operation, which would have likely provided him with no symptomatic improvement. While the cost-savings from such cases as the ones we presented are difficult to predict at a population level, we believe that they would far outweigh any additional cost incurred by implanting an MRI-compatible device. The other cost concern is that changing a pacemaker to an MR-compatible version is not a diagnostic code or trigger for insurance companies to approve the procedure. We hope that examples like these will shed light on the importance of placing the devices de novo and the flexibility of changing the pacemakers as the clinical scenarios warrant.

## Conclusions

Considering the importance of MR imaging in neurosurgical patients along with the disproportionate disease burden in the population of patients with pacemakers, MRI-compatible pacemakers offer a clear benefit. The four cases presented in this paper all provide evidence of instances where MRI findings significantly altered the patients' clinical course. In the cases where patients have an existing non-MRI-compatible system, the benefits of replacement must be carefully weighed against the risks.
